# Short Term Outcomes of Open and Minimally Invasive Approaches to Segmental Colectomy for Benign Colovesical Fistula

**DOI:** 10.1155/2022/9242813

**Published:** 2022-11-23

**Authors:** Zhobin Yeganeh, Dustin Hau Huynh, Anthony Paul Kopatsis, Anthony Kopatsis

**Affiliations:** ^1^Department of Trauma and Surgical Critical Care, Hospital Corporation of America (HCA) Florida, Osceola Hospital, Kissimmee, FL, USA; ^2^Department of Surgery, NYC Health and Hospitals-Elmhurst, New York, USA

## Abstract

**Background:**

We speculated that a minimally invasive (MIS) colectomy for colovesical fistula is associated with less morbidity compared to an open colectomy.

**Methods:**

Multivariate analysis using logistic regression was used to investigate the outcomes of patients who underwent colectomy for benign colovesical fistula during 2012–2017 by surgical approach using the NSQIP database.

**Results:**

We identified 748 patients underwent partial colectomy for benign colovesical fistula during 2012–2017. Surgeons used the MIS approach in 72.7% of operations, with a conversion rate of 13.1%. The MIS approach was associated with lower morbidity (27.4% vs. 43.1%, AOR: 0.46, *P*=0.02) compared to the open approach. The mean operation duration was longer in MIS operations compared to open (225 min vs. 201 min, *P* < 0.01). The robotic approach to colectomy showed no significant difference in morbidity (28.4% vs. 27.2%, *P*=0.77) but a decrease in conversion rate (8.1% vs. 13.8%, *P* < 0.01) and an increase in operation length (249 min vs. 222 min, mean difference: 27 min, *P* < 0.01) compared to a laparoscopic approach. There was no significant difference in the anastomotic leak rate between MIS and open approaches (3.7% vs. 5.4%, *P*=0.14) and between laparoscopic and robotic approaches (2.8% vs. 3.8%, *P*=0.99).

**Conclusions:**

We found a 72.7% utilization rate of MIS approach to colectomy for benign colovesical fistula in the NSQIP hospitals with a 13.6% conversion rate. Patients with MIS approach had significantly lower morbidity compared to open. A robotic approach to partial colectomy has the same morbidity risk with a decreased conversion rate compared to laparoscopic approach.

## 1. Introduction

Colovesical fistula is a condition that can be a complication of a variety of diseases and conditions, including diverticulitis, cancer, Crohn's disease, and radiation [1, 2]. Complicated diverticulitis with direct extension of a ruptured diverticulum or erosion of a peri-diverticular abscess into the bladder has been reported to be the most common cause, accounting for 70% of cases, followed by Crohn's disease in 10% of cases [1–3]. The fundamental principle of surgical management of colovesical fistula is removal of the fistula and diseased segment of colon [2, 4]. Morbidity of open procedures can be as high as 49% with a significant reoperation rate (up to 17%) [4–7]. Surgical treatment for colovesical fistula is evolving to decrease morbidity for the patients through the utilization of minimally invasive (MIS) approaches [4–7].

Feasibility, safety, and advantages of the MIS approach compared to a traditional open colectomy for diverticulitis have been previously cited [8–10]. A majority of patients undergoing an elective colectomy for diverticular disease receive a minimally invasive operation in the US now [8, 10]. Recently published data revealed that laparoscopic treatment of complicated diverticulitis with colovesical fistula is feasible and safe, with better outcomes compared to open surgery when performed by skilled laparoscopic surgeons [4, 11, 12]. However, considering the heterogeneous minimally invasive surgery (MIS) skills of surgeons, such conclusions can only be generalized if similar outcomes can be found in a larger study such as a national database study. Using a nationwide database, this study aims to compare 30 days complications of the MIS approach with the traditional open approach for elective nonmalignant colovesical fistula.

## 2. Methods

We performed a retrospective analysis using the American College of Surgeons National Surgical Quality Improvement Program (ACS-NSQIP) database for the years 2012–2017. We queried adult patients (age eighteen-year-old and more) who underwent partial colectomy for colovesical fistula whose data were submitted to the ACS NSQIP using the Participant Use Data Files (PUF) and the target colectomy files during 1/1/2012 to 12/31/2017. The NSQIP database is a nationally validated, prospective, multiinstitutional database extracted from medical records by trained surgical clinical reviewers. ACS-NSQIP details more than 300 data points for deidentified cases including patient demographics, comorbidities, perioperative characteristics, and 30-day postoperative complications in more than 600 participating institutions of varying sizes and academic affiliations [13]. All data points are from deidentified cases and the database is fully anonymized by the American College of Surgeons, and this study is exempt from IRB approval [13]. The NSQIP database is available for researchers nationwide at participating hospitals [13].

In this study, we selected adult patients with colovesical fistula who underwent partial colectomy with anastomosis based on the current procedural terminology (CPT) codes of 44140 and 44204 for open and MIS approaches, respectively. Patients who had cancer as the reason of colovesical fistula were excluded from the study. Patients with colovesical fistula were identified with an International Classification of Diseases, Ninth Revision (ICD-9) diagnosis code of 596.1 or International Classification of Diseases, Tenth Revision (ICD-10) diagnosis code of N32.1 within the database. The patients were separated into groups based on whether they underwent open or MIS approaches. Variables compared between groups included demographics (age, race, and gender), comorbidities (such as diabetes, hypertension, and so on.), operative factors (such as operation length and surgical approach), and outcomes (postoperative complications, mortality, hospitalization length, and so on.). The endpoints were comparing 30 days mortality and morbidity of the patients by surgical approaches. Variables in this study were defined as mentioned by the NSQIP User Guide, which can be referenced for detailed variable definitions online [13]. Overall morbidity is defined as the presence of at least one postoperative complication of anastomosis leakage, intra-abdominal infection, sepsis, septic shock, ventilator dependency, cardiac arrest, hemorrhagic complication needs transfusion, pulmonary embolism, myocardial infarction, pneumonia, central vascular accident, acute renal failure, progressive renal insufficiency, superficial surgical site infection, deep surgical site infection, unplanned reoperation, deep venous thromboembolism, urinary tract infection, unplanned intubation, prolonged ileus, and wound disruption. Severe morbidity was defined as the presence of at least one of the complications of cardiac arrest, intra-abdominal infection, septic shock, pulmonary embolism, ventilator dependency, acute renal failure, myocardial infarction, anastomosis leakage, and pneumonia.

### 2.1. Statistical Analyses

All analyses were performed with the Statistical Package for Social Sciences (SPSS) software, Version 22 (SPSS Inc., Chicago, IL). Patients were divided per surgical approaches (open vs. MIS) into two groups of patients. Comparisons of patient characteristics were performed using a chi-square test for categorical variables and an independent *t* test for continuous variables to determine the difference in proportions for dichotomous and categorical variables between groups in the study. All independent variables that showed a significant difference (0.05) in the univariate model were placed in multivariate logistic regression or linear regression models to identify independent risk factors for primary adverse outcomes. The adjusted odds ratio (AOR) and its confidence interval (CI) and *P* value were obtained from the final model as a measure of the association between the independent predictors and the dependent responses. The one-way analysis of variance was used to assess the difference in mean for continuous variables. A *P* value of <0.05 was considered to indicate a statistically significant difference for all statistical tests.

## 3. Results

A total of 748 patients who underwent open or MIS partial colectomy with anastomosis with a diagnosis of colovesical fistula during 1/1/2012–12/31/2017 were identified within the NSQIP database. Most patients were Caucasian (78.3%) and male (63.8%). The most prevalent comorbidities included hypertension (56.4%) and obesity (40.2%). Patients were divided per surgical approach (open vs. MIS) into two groups of patients. The descriptive statistics and patient demographics by surgical approach have been summarized in Table 1. Open surgery was more commonly performed in patients with COPD, weight loss, hypoalbuminemia, and renal failure on dialysis. There was limited information for type of bladder repair and using muscle flap to cover the fistula site. Three patients required partial cystectomy following resection of the colovesical fistula, and the rest of the patients had repair of bladder following colovesicular fistula without resection. Also, 25 patients had a report for an omental or muscle flap to cover the repaired site of the bladder.

Overall, 544 (72.7%) of operations were conducted with MIS approach. Of these 74 patients (13.6%) had robotic surgery. Overall conversion rate to open for MIS approach was observed in 13.1% of cases (8.1% for robotic and 13.8% for laparoscopic approach). There was a steady increase in the utilization of the MIS approach to colovesical fistula from 63.9% for 2012 to 78.8% for 2017. The median postoperative hospitalization length for MIS and open approaches to colovesical fistula were 5 and 8 days, respectively. The mean operation duration was longer in MIS approach compared to open operations (225 min vs. 201 min, *P* < 0.01). Also, the mean operation duration was longer in robotic approach compared to laparoscopic operations (249 min vs. 221 min, *P* < 0.01).

A risk adjusted analysis of factors associated with 30-day mortality and morbidity in the patients with colovesical fistula who underwent partial colectomy with anastomosis are reported in Tables 2 and 3. Although surgical approach was not associated with mortality of the patients (AOR: 1.71, *P*=0.59), MIS approach was significantly associated with decreased morbidity (AOR: 0.49, *P*=0.01). Also, the American Society of Anesthesiologists (ASA) score more than two was significantly associated with an increase in morbidity of the patients (AOR; 2.05, *P*=0.01).

30-day mortality, overall morbidity, severe morbidity, and postoperative complications of patients who underwent partial colectomy with anastomosis for colovesical fistula per surgical approach have been reported in Table 4. Overall morbidity, severe morbidity, sepsis, and hemorrhagic complications were significantly lower in the MIS approach compared to the open approach (Table 4).

A risk adjusted analysis of postoperative complications of the patients with colovesical fistula who underwent partial colectomy with anastomosis with the laparoscopic and robotic approaches are reported in Table 5. Multivariate analysis revealed there was not any significant difference in 30-day postoperative complications between the robotic and laparoscopic approaches. Conversion to open was higher in laparoscopic approach compared to robotic approach (13.8% vs. 8.8%, *P* < 0.01). However, the mean operation duration was longer in robotic approach than laparoscopic operations (248 min vs. 221 min, *P* < 0.01). When comparing anastomosis leakage for patients who underwent MIS approach there was no significant difference in the anastomotic leak rate of intracorporeal versus extracorporeal anastomosis (4.2% vs. 3.8%, AOR: 1.44, *P*=0.70).

## 4. Discussion

Our study results show minimally invasive approach to colovesical fistulas is associated with less 30-day morbidity and shorter hospitalization length compared to open approach. We found a decrease in overall and severe morbidity of MIS approach compared to open with no significant change in mortality risk. Diverticular fistula is not a contraindication for the MIS approach, and multiple recently published articles revealed the benefits of MIS approach to colovesical fistula [4, 12, 14–18]. Along this line we found an increase in utilization of MIS approach to colovesical fistulas during 2012–2017. Although American Society of Colorectal Surgeons (ASCRS) text book for colon and rectum surgery mentioned the benefits of the MIS approach for complicated diverticulitis compared to noncomplicated diverticulitis (page 660 chapter 39), the lack of RCTs does not allow for the drawing of statistically significant conclusions on the MIS approach for colovesical fistulas, despite the fact that this approach is considered safe [19].

We found a significant decrease in the risks of overall morbidity, severe morbidity, sepsis, and hemorrhagic complications using the MIS approach compared to open surgery. In addition, we found the MIS approach is associated with shorter hospitalization compared to open surgery. Also, our study results show that there was a trend toward a decrease in multiple other complications in the MIS group compared to the open group that did not reach the level of statistical significance (Table 4). The benefits of the MIS approach in colorectal surgery have been discussed broadly in the literature [8, 10, 20, 21]. However, comparing the open and MIS approaches may be confounded by selection bias as the baseline characteristics of the two groups of patients with the MIS and open approaches in this study were heterogeneous (Table 1). We found that patients who underwent the MIS approach had less comorbid conditions of COPD, weight loss, and preoperative sepsis. This finding shows there is a trend to operate with an open approach for sicker patients, which might be due to the shorter operation length. Prospective clinical trials need to compare outcomes of the open and MIS approaches to colovesicular fistula in two homogeneous groups of patients.

We found that the robotic approach to colovesical fistula may have advantages over the laparoscopic approach. When comparing the laparoscopic approach to the robotic approach, we could not find any significant differences in morbidity and mortality risks. However, there was a trend toward a decrease in severe morbidity of robotic approach compared to the laparoscopic approach that did not reach the level of statistical significance in multivariate analysis. Also, the robotic approach had a significantly less conversion rate to open compared to laparoscopic approach in this study (8.1% vs. 13.8%). Features of robotic surgery such as three-dimensional vision, restoration of the eye-hand-target axis, better depth perception, and a better definition of tissue planes that leads to precise dissection can be factors that help overcome some of the challenges of laparoscopic surgery and lead to a decrease in the conversion rate [22, 23]. The benefits of a robotic approach compared to the laparoscopic approach must be weighed against the longer operation length. More research is needed to better understand if the longer operation and probably increased cost in robotic approach is justified by an improvement in outcomes.

We found longer operation times with shorter hospitalization for the patients underwent the MIS approach to colovesical fistula compared to open surgery. Shorter hospitalization length is one of the general advantage of the MIS approach which can result in significant reduce in costs per patient [10, 24–26]. However, the benefits of a MIS approach must be weighed against the longer operation time. Advancements in dissection and coagulation devices and increased experience of surgeons in MIS surgery may decrease the length of the procedures [23, 27]. However, selected cases who cannot tolerate carbon dioxide insufflation for long periods of time may still benefit from an open approach [28].

## 5. Study Limitations

This study has some limitations. This is a retrospective study, and we are unable to draw any causal conclusions and our study results need to be confirmed with a prospective randomized control trial. We could investigate the 30-day postoperative complications of the patients who had operations for colovesical fistula. However, information on the long term outcomes of the patients was not available in the NSQIP database. We compared two groups of patients who had an open and the MIS approach to colovesical fistula. However, the baseline characteristics of these two groups of patients were heterogeneous, and any conclusions may have bios. We attempted to adjust the results for all possible confounders, we could not capture all potentially important explanatory variables such as details of the surgical procedure, reason of conversion to open, and previous abdominal operation. There was limited information for type of bladder repair as well as to use of a muscle flap to cover the repaired site due to coding limitation and we could not compare the type of bladder repair (with or without resection) and the benefit of a muscle flap to prevent relapse of the fistula in our study. Thirty despite these limitations, the advantage of using the NSQIP database is the broad national geographic representation across all regions of the country with different surgeons MIS skills and this makes it a suitable database to evaluate outcomes in not just tertiary referral centers with specialized surgeons with high MIS skills but a great variety of centers.

## 6. Conclusions

The majority of segmental colectomies for benign colovesical fistula in the NSQIP hospitals are being performed with the MIS approach (72.7%). Our study result shows patients with colovesical fistula who were treated with MIS approaches had significantly lower morbidity compared to an open approach. In the majority of the cases, colonic anastomosis in the MIS approach is done with the intracorporeal technique (53.4%) without a significant change in the risk of anastomotic leak compared to extracorporeal anastomosis. The robotic approach to benign colovesical fistula happened in 13.6% of total MIS cases with the same morbidity risk with a modestly decreased conversion rate compared to the laparoscopic approach. Based on these results an MIS approach should be utilized when possible.

## Figures and Tables

**Table 1:** Demographics and clinical characteristics of patient population of the study by surgical approach.

Variables		Minimally invasive approach = 544	Open approach = 204	*P* values
Age	Age more than 70 years	150 (27.8%)	60 (29.4%)	0.65
Sex	Female	183 (33.6%)	88 (43.1%)	0.01
Race	White	436 (87.6%)	150 (82%)	0.04
	Black or African American	43 (8.6%)	19 (10.4%)	0.04
	Asian	11 (2.2%)	11 (6%)	0.01
	Other	8 (1.6%)	3 (1.6%)	0.98
Comorbidity	Diabetes mellitus	88 (16.2%)	24 (11.8%)	0.13
	Weight loss	14 (2.6%)	17 (8.3%)	<0.01
	Congestive heart failure	4 (0.7%)	3 (1.5%)	0.35
	Chronic steroid use	40 (7.4%)	13 (6.4%)	0.64
	Chronic obstructive pulmonary disease	37 (6.8%)	25 (12.3%)	0.01
	Smoking	102 (18.8%)	54 (26.5%)	0.02
	Moderate or severe dyspnea	36 (6.6%)	16 (7.8%)	0.55
	Renal failure on dialysis	2 (0.4%)	4 (2%)	0.03
	Hypertension	310 (57%)	112 (54.9%)	0.60
Body mass index	<30	329 (60.7%)	116 (57.4%)	0.03
	30–39.9	109 (20.1%)	38 (18.8%)	0.03
	≥40	104 (19.2%)	48 (23.8%)	0.01
Other factors	Mean operation length	225 min	201 min	<0.01
	Preoperative sepsis	6 (1.1%)	7 (3.4%)	0.03
	Mechanical bowel preparation	366 (78.7%)	112 (70.4%)	0.03
	Chemical bowel preparation	258 (53.1%)	85 (49.4%)	0.40
	Preoperative leukocytosis > 10,000 mm^3^	108 (21.3%)	41 (21.2%)	0.98
	ASA^*∗*^score more than two	295 (54.2%)	120 (58.8%)	0.26
	Serum albumin level less than 3.5 g/dL	53 (16.8%)	39 (30.5%)	<0.01

^*∗*^The American Society of Anesthesiologists score.

**Table 2:** Multivariate analysis of factors associated with mortality of patients with colovesical fistula who underwent operation.

Variables		Adjusted odd ratio	95% confidence interval	*P* values
Age	Age > 70 years	5.68	0.58–55.33	0.13
Sex	Female	1.47	0.17–12.23	0.72
Comorbidity	End stage renal disease on dialysis	1.01	0.90–1.10	0.99
	Congestive heart failure	1.02	0.89–1.11	0.99
	Moderate or severe dyspnea	1.01	0.91–1.11	0.99
	Chronic obstructive pulmonary disease	7.03	0.55–89.43	0.13
	Hypertension	0.15	0.005–4.86	0.28
	Smoking	0.53	0.03–7.75	0.64
	Diabetes	1.01	0.90–1.10	0.99
	Hypertension	0.71	0.06–6.24	0.65
	Chronic steroid use	3.52	0.22–56.42	0.37
	Weight loss	18.14	0.46–702.55	0.12
Body mass index	<30	Reference	Reference	—
	30–39.9	1.02	0.90–1.12	0.99
	40≤	1.01	0.92–1.11	0.99
Other factors	Operation longer than 3 hours	0.92	0.90–1.10	0.99
	Mechanical bowel preparation	0.18	0.02–1.45	0.10
	The American Society of Anesthesiologists (ASA) score more than two	1.01	0.90–1.11	0.99
	Preoperative leukocytosis > 10,000 mm^3^	1.17	0.08–16.42	0.90
	Preoperative sepsis	1.10	0.98–1.12	0.99
	Serum albumin less than 3 g/dL	1.45	0.17–12.41	0.73
	Chemical bowel preparation	0.41	0.02–5.92	0.51
	Minimally invasive approach vs. open	1.71	0.23–12.27	0.59

**Table 3:** Multivariate analysis of factors associated with morbidity of patients with colovesical fistula who underwent operation.

Variables		Adjusted odd ratio	95% confidence interval	*P* values
Age	Age > 70 years	1.07	0.0.57–1.99	0.83
Sex	Female	1.50	0.86–2.60	0.14
Comorbidity	End stage renal disease on dialysis	1.01	0.90–1.10	0.99
	Congestive heart failure	0.79	0.10–6.24	0.82
	Moderate or severe dyspnea	1.51	0.47–4.77	0.48
	Chronic obstructive pulmonary disease	1.33	0.52–1.39	0.54
	Hypertension	1.26	0.67–2.38	0.46
	Smoking	0.53	0.03–7.75	0.64
	Diabetes	1.64	0.81–1.31	0.16
	Hypertension	0.96	0.54–1.71	0.91
	Chronic steroid use	0.48	0.17–1.34	0.16
	Weight loss	0.40	0.10–1.52	0.18
Body mass index	<30	Reference	Reference	—
	30–39.9	1.63	0.84–3.17	0.14
	40≤	0.93	0.63–1.36	0.71
Other factors	Operation longer than 3 hours	0.90	0.31–2.58	0.84
	Mechanical bowel preparation	0.77	0.41–1.45	0.42
	The American Society of Anesthesiologists (ASA) score more than two	2.05	1.15–3.63	0.01
	Preoperative leukocytosis > 10,000 mm^3^	1.17	0.63–2.20	0.60
	Preoperative sepsis	14.01	1.56–125.48	0.01
	Serum albumin less than 3 g/dL	1.17	0.63–2.17	0.61
	Chemical bowel preparation	0.90	0.52–1.53	0.70
	Minimally invasive approach vs. open	0.49	0.27–0.87	0.01

**Table 4:** Postoperative 30 days complications of patients with colovesical fistula by surgical approach (open vs. minimally invasive).

Complications	Minimally invasive approach total = 544	Open approach total = 204	Adjusted odd ratio (confidence interval)	*P* values
Mortality	6 (1.1%)	5 (2.5%)	1.71 (0.23–12.27)	0.59
Overall morbidity^*∗*^	149 (27.4%)	88 (43.1%)	0.49 (0.27–0.87)	0.01
Severe morbidity^*∗∗*^	54 (10%)	43 (21.1%)	0.57 (0.31–0.90)	0.04
Ventilator dependency more than 48 hours	7 (1.3%)	7 (3.4%)	0.17 (0.008–3.60)	0.25
Prolonged ileus	64 (11.8%)	32 (15.8%)	0.50 (0.22–1.15)	0.10
Unplanned intubation	8 (1.5%)	9 (4.4%)	0.26 (0.01–6.12)	0.40
Pneumonia	9 (1.7%)	7 (3.4%)	0.07 (0.002–3.74)	0.19
Intra-abdominal infection	33 (6.1%)	17 (8.3%)	0.57 (0.19–1.70)	0.31
Superficial surgical site infection	20 (3.7%)	12 (5.9%)	0.46 (0.13–1.62)	0.23
Deep surgical site infection	1 (0.2%)	2 (1%)	0.98 (0.90–1.02)	0.99
Wound disruption	2 (0.4%)	4 (2%)	1.01 (0.91–1.11)	0.99
Sepsis	22 (4%)	20 (9.8%)	0.26 (0.08–0.80)	0.01
Septic shock	9 (1.7%)	7 (3.4%)	1.01 (0.90–1.10)	0.99
Hemorrhagic complications need transfusion	24 (4.4%)	25 (12.3%)	0.29 (0.10–0.83)	0.02
Unplanned reoperation	26 (4.8%)	21 (10.3%)	0.30 (0.07–1.28)	0.10
Deep venus thromboembolism	9 (1.7%)	2 (1%)	0.34 (0.03–3.15)	0.34
Pulmonary embolism	4 (0.7%)	1 (0.5%)	1.10 (0.90–1.19)	0.98
Central vascular accident (CVA)	0	1 (0.5%)	^*∗∗∗*^	^*∗∗∗*^
Cardiac arrest requiring cardiopulmonary resuscitation	1 (0.2%)	2 (1%)	1.01 (0.98–1.10)	0.99
Anastomosis leakage	20 (3.7%)	11 (5.4%)	0.31 (0.06–1.45)	0.14
Acute renal failure	5 (0.9%)	2 (1%)	1.01 (0.90–1.10)	0.98
Urinary tract infection	38 (7%)	18 (8.8%)	1.64 (0.57–4.76)	0.35
Progressive renal insufficiency	0	1 (0.5%)	^*∗∗∗*^	^*∗∗∗*^
Myocardial infarction	3 (0.6%)	2 (1%)	1.02 (0.90–1.10)	0.99

^*∗*^includes: intra-abdominal infection, sepsis, septic shock, ventilator dependency, cardiac arrest, acute renal failure, hemorrhagic complication needs transfusion, pulmonary embolism, myocardial infarction, pneumonia, central vascular accident, superficial surgical site infection, deep surgical site infection, unplanned reoperation, deep venus thromboembolism, urinary tract infection, progressive renal insufficiency, unplanned intubation, anastomosis leakage, prolonged ileus, and wound disruption.

**Table 5:** Postoperative 30 days complications of patients with colovesical fistula by surgical approach (laparoscopic vs. robotic).

Complications	Robotic approach total = 74	Laparoscopic approach total = 470	Adjusted odd ratio (confidence interval)	*P* values
Mortality	1 (1.4%)	5 (1.1%)	0.26 (0.19–1.12xcd)	0.98
Overall morbidity^*∗*^	21 (28.4%)	128 (27.2%)	0.87 (0.34–2.19)	0.77
Severe morbidity^*∗∗*^	3 (4.2%)	51 (10.9%)	0.11 (0.01–1.20)	0.07
Ventilator dependency more than 48 hours	1 (1.4%)	6 (1.3%)	1.01 (0.90–1.12)	0.99
Prolonged ileus	8 (10.8%)	56 (11.9%)	1.29 (0.34–4.80)	0.70
Unplanned intubation	2 (2.7%)	6 (1.3%)	2.69 (0.70–3.11)	0.99
Pneumonia	1 (1.4%)	8 (1.7%)	0.98 (0.88–1.12)	0.99
Intra-abdominal infection	2 (2.7%)	31 (6.6%)	0.90 (0.80–1.20)	0.99
Superficial surgical site infection	2 (2.7%)	18 (3.8%)	4.52 (0.30–67.99)	0.27
Deep surgical site infection	0	1 (0.2%)	^*∗∗∗*^	^*∗∗∗*^
Wound disruption	0	2 (0.4%)	^*∗∗∗*^	^*∗∗∗*^
Sepsis	1 (1.4%)	21 (4.5%)	0.90 (0.80–1.20)	0.99
Septic shock	1 (1.4%)	8 (1.7%)	0.92 (0.82–1.10)	0.97
Hemorrhagic complications need transfusion	2 (2.7%)	22 (4.7%)	0.78 (0.03–12.82)	0.66
Unplanned reoperation	2 (2.7%)	24 (5.1%)	0.94 (0.88–1.10)	0.98
Deep venus thromboembolism	1 (1.4%)	8 (1.7%)	0.89 (10.80–1.03)	0.99
Pulmonary embolism	0	4 (0.9%)	^*∗∗∗*^	^*∗∗∗*^
Central vascular accident (CVA)	0	0	^*∗∗∗*^	^*∗∗∗*^
Cardiac arrest requiring cardiopulmonary resuscitation	0	1 (0.2%)	^*∗∗∗*^	^*∗∗∗*^
Anastomosis leakage	2 (2.8%)	18 (3.8%)	0.90 (0.88–1.07)	0.98
Acute renal failure	0	5 (1.1%)	^*∗∗∗*^	^*∗∗∗*^
Urinary tract infection	7 (9.5%)	31 (6.6%)	1.25 (0.33–4.62)	0.75
Progressive renal insufficiency	0	0	^*∗∗∗*^	^*∗∗∗*^
Myocardial infarction	0	3 (0.6%)	^*∗∗∗*^	^*∗∗∗*^

^*∗*^includes: intra-abdominal infection, sepsis, septic shock, ventilator dependency, cardiac arrest, acute renal failure, hemorrhagic complication needs transfusion, pulmonary embolism, myocardial infarction, pneumonia, central vascular accident, superficial surgical site infection, deep surgical site infection, unplanned reoperation, deep venus thromboembolism, urinary tract infection, progressive renal insufficiency, unplanned intubation, anastomosis leakage, prolonged ileus, and wound disruption. ^*∗∗*^ includes: intra-abdominal infection, septic shock, ventilator dependency, cardiac arrest, acute renal failure, pulmonary embolism, myocardial infarction, pneumonia, unplanned reoperation, anastomosis leakage, and unplanned intubation. ^*∗∗∗*^ at least in one group of the patients there was not any case to compare.

## Data Availability

The data used to support the findings of this study are available at the national database NSQIP.
